# The Design, Fabrication, and Evaluation of a Phase-Resolved Partial Discharge Sensor Embedded in a MV-Class Bushing

**DOI:** 10.3390/s23249844

**Published:** 2023-12-15

**Authors:** Gyeong-Yeol Lee, Nam-Hoon Kim, Dong-Eon Kim, Gyung-Suk Kil, Sung-Wook Kim

**Affiliations:** 1 Department of Electrical and Electronics Engineering, Korea Maritime and Ocean University, Busan 49112, Republic of Korea; priority1@g.kmou.ac.kr (G.-Y.L.); dom0404@g.kmou.ac.kr (N.-H.K.); jklfds1003@g.kmou.ac.kr (D.-E.K.); 2 Department of Electrical and Electronics Engineering, Silla University, Busan 46958, Republic of Korea; number1@silla.ac.kr

**Keywords:** PRPD sensor, MV-class bushing, accuracy class, phase error, partial discharges

## Abstract

This paper proposes a novel phase-resolved partial discharge (PRPD) sensor embedded in a MV-class bushing for high-accuracy insulation analysis. The design, fabrication, and evaluation of a PRPD sensor embedded in a MV-class bushing aimed to achieve the detection of partial discharge (PD) pulses that are phase-synchronized with the applied primary HV signal. A prototype PRPD sensor was composed of a flexible printed circuit board (PCB) with dual-sensing electrodes, utilizing a capacitive voltage divider (CVD) for voltage measurement, the D-dot principle for PD detection, and a signal transducer with passive elements. A PD simulator was prepared to emulate typical PD defects, i.e., a metal protrusion. The voltage measurement precision of the prototype PRPD sensor was satisfied with the accuracy class of 0.2 specified in IEC 61869-11, as the maximum corrected voltage error ratios and corrected phase errors in 80%, 100%, and 120% of the rated voltage (13.2 kilovolts (kV)) were less than 0.2% and 10 min, respectively. In addition, the prototype PRPD sensor had good linearity and high sensitivity for PD detection compared with a conventional electrical detection method. According to performance evaluation tests, the prototype PRPD sensor embedded in the MV-class bushing can measure PRPD patterns phase-synchronized with the primary voltage without any additional synchronization equipment or system. Therefore, the prototype PRPD sensor holds potential as a substitute for conventional commercial PD sensors. Consequently, this advancement could lead to the enhancement of power system monitoring and maintenance, contributing to the digitalization and minimization of power apparatus.

## 1. Introduction

Insulation degradation in power equipment can be predicted by detecting partial discharge (PD) pulses in the early stages. Various PD sensors have been adapted to detect PD pulses [1,2], including a coupling capacitor, employing the conventional method based on IEC 60270 [3], and a high-frequency current transformer (HFCT), ultra-high-frequency (UHF) sensor, and an acoustic emission (AE) sensor, based the non-conventional method. The conventional detection method produces high-precision PD measurements and shows the output in picocoulomb (pC) by applying external voltage sources. However, it has the disadvantages of requiring the installation of a coupling capacitor for quantitative measurements, being unusable during operation, and limits on-site PD measurements to a maximum measurement frequency of 1 megahertz (MHz) [4,5,6,7]. On the other hand, UHF sensors have several advantages, including high sensitivity, good signal-to-noise ratio (S/N), high frequency range (300 kilohertz (kHz) to 3 gigahertz (GHz)), ability to estimate fault location, and continuous monitoring. Despite these advantages, UHF measurements have the disadvantage of being unable to calibrate the output magnitude in a unit of pC and are expensive [8,9,10]. AE sensors are widely used to detect internal defects in electrical power equipment owing to their cheap price and easy installation. The internal fault location can be estimated by calculating the amplitudes and different arrival times of several AE sensors. It cannot be measured in terms of pC, much like with UHF sensors, and the effects of reflections, attenuations, and the scattering of acoustic waves due to the internal structures of the equipment must be considered [11,12,13,14,15]. The above PD sensors are selected based on installation conditions and purpose. The most suitable method for insulation diagnosis is phase-resolved partial discharge (PRPD) analysis, which includes the phase angle (∅), PD magnitude (q), and the number of PD pulses (n) over a period within one cycle of the applied voltage source [16,17].

An accurate measurement of system voltage signals is critical to improve the safety and reliability of power equipment. The voltage signals obtained with various instruments, including iron-core-type potential transformers (PTs), capacitive potential transformers (CPTs), and resistive potential transformers (RPTs), have a critical role in the operation of protective relays to counter abnormal voltage surges. Iron-core-type PTs require a significantly large installation space due to their iron core and copper wire components, and can be susceptible to external transients when connected directly between primary and secondary circuits [18]. Capacitive potential transformers must be connected to high-input impedance instruments, typically exceeding several megohms. Alternatively, an impedance transformer must be used to match the input and output impedance between the CPT and the instrument. While these instruments can be used effectively within a narrow frequency band corresponding to commercial frequencies, their accuracy can be compromised if the voltage signal is contaminated with high-frequency noise components [19,20]. Therefore, the development of high-precision voltage measurement instruments with broadband frequency capability is essential. To address the challenges associated with ensuring an adequate insulation distance for direct connections to primary high-voltage conductors, as well as issues such as magnetic saturation, the deformation of internal cores, and the need for significant installation space, a novel voltage measurement method for low-power voltage transformers (LPVTs) has been the subject of recent research. This need prompted the International Electrotechnical Commission (IEC) to publish IEC 61869-11 [21], relevant to low-power voltage transformers (LPVTs) using passive elements, to replace IEC 60044-7 [22], which is currently applied to electronic voltage transformers (EVTs). This is intended for connection to stand-alone merging units (SAMUs) or metering devices according to IEC 61869-13 [23].

Wagoner et al. [24] diagnosed the current and voltage output signals in the vacuum section of a 20-mega-ampere (MA) 3-megavolt (MV) pulsed-power accelerator using differential D-dot and B-dot sensors with a common mode for noise rejection. Wang and colleagues [25] developed voltage transformers using the basis of a differential D-dot sensor. They experimented and simulated the designed D-dot probe sensor for the verification of measurement accuracy. Kim et al. [26] developed an electronic voltage transformer (EVT) with an accuracy class of 0.2 using a D-dot sensor. They showed that the prototype EVT can accurately detect voltage signals up to the third, fifth, and seventh harmonics at a commercial frequency of 60 hertz (Hz) upon employing a non-contact voltage measurement method.

Wang and colleagues [27] investigated an electronic voltage transformer with a self-integral D-dot sensor using the D-dot principle for high-voltage signal measurement. They found that the D-dot sensor operates self-integrated modes with excellent phase frequency characteristics by applying parallel and differential structures of multiple electrodes. Yao and colleagues [28] proposed a compensation method that improves the accuracy of output signals by minimizing the offset due to the integrated circuit of the D-dot electric field sensor. A mathematical method from this study was proposed to reduce the offset value by the integration circuit. However, in the view of condition monitoring, the proposed devices and methods cannot detect abnormal pulses from internal defects because they are mainly designed to measure system voltage signals.

Hussain et al. [29] studied an online monitoring sensor, capturing abnormal electrical fault signals generated from an internal arc for medium-voltage (MV) switchgears based on the differential D-dot principle. Hussain and colleagues [30] compared the detection characteristics of a Rogowski coil, loop antenna, HFCT, and D-dot sensor in air-insulated switchgears, and discovered that a Rogowski coil sensor and D-dot sensor are more suitable for PD measurements due to their high S/N. Rostaghi-Chalaki and colleagues [31] investigated the output characteristics of a D-dot and B-dot measuring DC PD pulses propagating through a transmission line (TL) using the electromagnetic (EM) field principle. They found that the apparent discharge measured by the EM field sensors was almost identical to the reference PD pulse measured with an oscilloscope. Jin and colleagues [32] studied the measurement of a transient-pulsed electromagnetic field using a D-dot sensor and outlined a compensation system for the recovery of the incident E-field to improve the dynamic characteristics.

Information about the phase distribution of PD pulses is essential for the PD diagnosis of high-voltage power equipment since the PRPD analysis method is typically used to identify types of PD defects for on-site PD measurement. However, acquiring the reference voltage signals directly from the power equipment in on-site operation is challenging and inconvenient. In addition, sometimes, the reference voltage signal is measured at a considerable distance from the expected PD defect location. In terms of PD fault identification, the diagnostic accuracy depends on the synchronization of the detected PD pulse with the phase of the reference voltage signal. Therefore, many studies have been conducted regarding how to obtain PD pulses phase-synchronized with the applied high voltage or the zero-crossing point of the applied high voltage. Kim et al. [33] suggested a possible diagnosis technique of unknown phase-shifted PD signals for GISs. The new diagnosis method utilized the shapes, distribution ranges, density, and peak values of the PD pulses and could classify internal defect types and noises without the phase distribution information of the applied voltage. Lee and colleagues [34] developed a neural network algorithm to discriminate phase-shifted PRPD patterns. They proposed a new method which was able to convert the fundamental phase-shifted parameters, such as phase angle, magnitude, and the number of PD pulses, to standardized parameters by applying the neural network algorithm method. However, there are limitations to setting criteria for determining internal defects, as their identification relies on the knowledge and experience of the engineer. Therefore, the development of techniques for the acquisition of accurate phase angles of applied voltage signals for insulation diagnosis remains necessary.

To address these limitations, this paper proposes a PRPD sensor embedded in a MV-class bushing, capable of detecting phase-synchronized PD pulses through precise measurements of the primary HV signal. The prototype PRPD sensor demonstrated a voltage measurement accuracy that was satisfied with an accuracy class of 0.2 by analyzing the error ratio and phase error according to the test guidelines in IEC 61869-11. Furthermore, the PRPD sensor was found to have good linearity and sensitivity in PD detection by comparing the output magnitude and PRPD pattern detected using the conventional electrical detection method specified in IEC 60270. It is expected that the prototype PRPD sensor can minimize the installation area of epoxy insulation and help to improve the precision of insulation diagnosis for acquiring PD pulses phase-synchronized with the applied signal.

## 2. Design and Fabrication

Detecting PD pulses phase-synchronized with the applied voltage signal is very important to identify and distinguish insulation defects. The prototype PRPD sensor embedded in a MV-class bushing consists of dual-sensing electrodes and signal transducers for voltage signal measurement and PD detection.

### 2.1. PRPD Sensor Embedded in a Bushing

**Figure 1:**
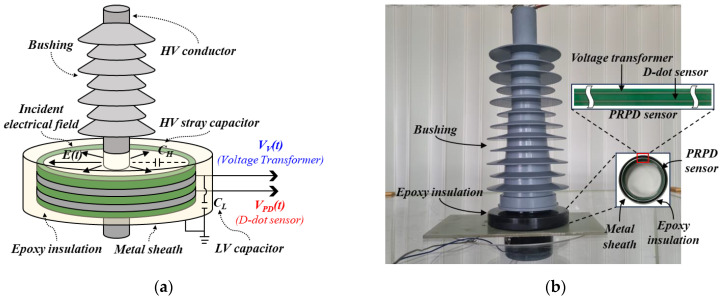
The prototype PRPD sensor: (**a**) configuration and (**b**) photograph.

Figure 1 shows a configuration and photograph of the prototype PRPD sensor embedded in a MV-class bushing. The PRPD sensor was designed using a non-contact detection method. The dual-sensing electrodes consisted of a voltage transformer (VT) for voltage signals with a commercial frequency band (around 60 Hz) and a D-dot sensor for PD pulses with high-frequency ranges, respectively. The electrodes were installed on a flexible PCB to encircle the HV conductor and minimize external environmental impacts such as shock or vibration. The main manufacturing process of PRPD sensors consists of the following steps: Initially, the PRPD sensor is designed and fabricated on a flexible PCB by calculating the geometric parameters and insulation distance. The PRPD sensor is then embedded within an epoxy-insulated metal enclosure to minimize the influence of unexpected external electric fields. Finally, the PRPD sensor, housed in a metal enclosure, is installed in a MV-class bushing.

The capacitive divider principle was applied for the design and fabrication of the VT sensor as this does not allow for derivative output signals according to IEC 61869-11. The output voltage of the VT, $V_{V}(t)$, is proportional to the primary HV signal, $U_{P}(t)$, and it can be calculated using Equation (1):

(1) $$V_{V}(t)=\frac{C_{H}}{C_{H}+C_{L}+C_{V}}\times U_{P}(t)$$

where $C_{H}$ is a HV stray capacitor between the HV conductor and the sensing electrode of the VT, $C_{L}$ is an LV capacitor between the sensing electrode and a grounded metal sheath, and $C_{V}$ is a capacitor for controlling the transformation ratio in parallel with the LV capacitor. For the study outlined in this paper, the rated transformation ratio of the prototype PRPD sensor was set to 10,000:1.

Contrastingly, in accordance with the Gaussian law [20,21,34], the output voltage of the D-dot sensor $V_{\mathrm{PD}}(t)$ is proportional to the primary derivation value of the incident electrical field, $\vec{E}\left(t\right)$, and can be calculated using Equation (2):

(2) $$V_{\mathrm{PD}}\left(t\right)=R_{m}\times S_{\mathrm{eq}}\times \varepsilon \times \frac{d\vec{E}\left(t\right)}{\mathrm{dt}}$$

where $R_{m}$ is an output impedance of $V_{\mathrm{PD}}$, $S_{\mathrm{eq}}$ is an equivalent area of the closed surface of the D-dot electrode, ε is the permittivity of an epoxy insulation, and $\vec{E}\left(t\right)$ is the magnitude of the incident electrical field generated by the HV conductor. Since the duration time of PD pulses is less than the range of a few to several hundreds of nanoseconds, they do not require the application of a restoration process.

Table 1 shows the geometric parameters of the prototype PRPD sensor outlined in this paper. A high-glass-transition-temperature (T_g_-type) PCB was used to prevent deformation by heat generated during the epoxy-molding process. The PRPD sensor was housed within an aluminum alloy metal sheath for protection from external electrical fields such as shocks, vibration, or surges. The new application of the PRPD sensor was designed and fabricated with advantages such as good linearity, high sensitivity, low manufacturing cost, and being installation location-agnostic.

The geometric parameters of diameter, height, width, and thickness of the sensing electrodes were calculated to ensure not only the output accuracy of the PRPD sensor but also a sufficient insulation distance from the HV conductor.

### 2.2. Signal Transducer

**Figure 2:**
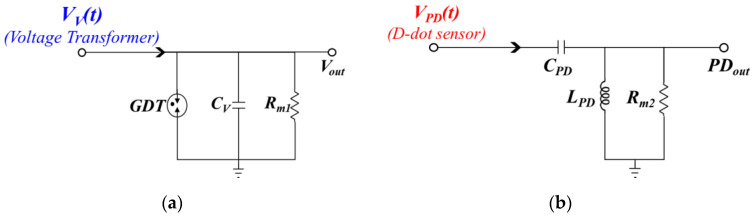
Configuration of the signal transducers: (**a**) voltage signals and (**b**) PD pulses.

The output signals from the VT and D-dot sensor of the PRPD sensor were connected with each signal transducer, as shown in Figure 2. In a signal transducer for the VT, $C_{V}$ as the transformation ratio control capacitor and $R_{m1}$ for impedance matching were installed in parallel with $C_{L}$, the LV capacitor of the VT. The magnitude of $C_{L}$ was fixed by the insulation material and geometric parameters including thickness, length, and width. Therefore, based on Equation (1), the magnitude of $C_{V}$ should be chosen carefully to satisfy the high-accuracy measurement specified in IEC 61869-11. The output resistance, $R_{m1}$, was set to 2 megaohm (MΩ) for impedance matching with the measuring instrument. A gas discharge tube (GDT) was installed at the front of the transducer circuit to protect from unexpected surges during the experiment.

On the other hand, in the signal transducer for the D-dot sensor, $C_{\mathrm{PD}}$ and $L_{\mathrm{PD}}$ were installed for operating as high-pass filters (HPFs) to obtain PD pulses with a high frequency range. The output resistor, $R_{m2}$, was set to 50 ohm (Ω) and was connected in parallel with $L_{\mathrm{PD}}$ for impedance matching with the measurement instrument.

**Figure 3:**
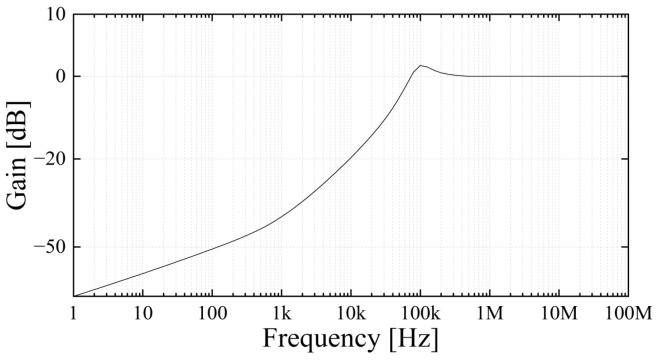
Frequency response of the signal transducer for the PD pulses.

Figure 3 shows the frequency response of the signal transducer for the PD pulses. Due to the propagation characteristics of the distributed elements of a conductor, the UHF component of PD pulses is attenuated by dielectric loss (tan δ) [35,36]. Therefore, the low cut-off frequency of the transducer was set about 63 kHz (−3 decibel (dB)). The gain was set to 1 for frequencies above 200 kHz, considering the attenuation of UHF band signals caused by the distributed element.

## 3. Experiment and Method

### 3.1. PD Simulator

PD pulses are important indicators of insulation deterioration analysis because they occur at an early stage inside electrical equipment. Therefore, internal defects can be predicted by detecting PD pulses before their breakdown. Despite the scientific efforts of manufacturers, installers, and operators to prevent the introduction of foreign objects and contaminants during the manufacturing, installation, and operation phases, small defects are still detected in electrical equipment. These defects can lead to insulation degradation.

Throughout the manufacturing process or during operation, the existence of imperfections or irregularities in materials, welding, or assembly can lead to the formation of sharp metal protrusions. When the electrical field is concentrated at the apex of these metal protrusions, it triggers a corona-type PD. Typically, PD pulses originate from a specific location on the metal protrusion, where it may be on an enclosure of electrical equipment.

**Figure 4:**
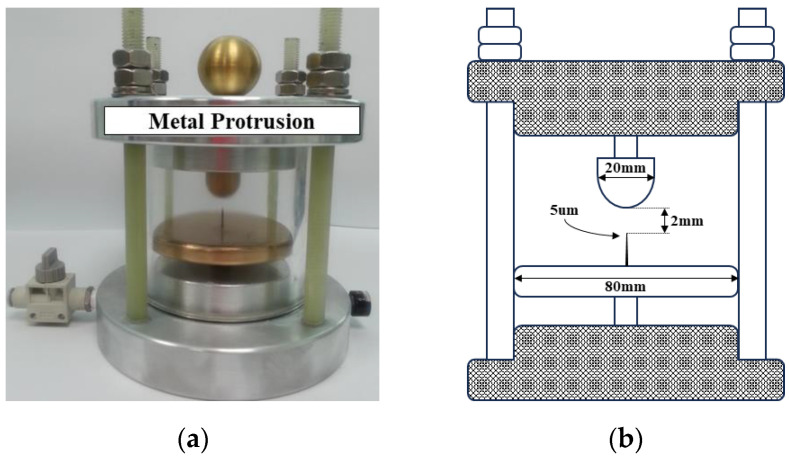
An artificial PD simulator: (**a**) photograph and (**b**) cross-sectional diagram.

Figure 4 shows the PD simulator of a metal protrusion defect. The metal protrusion was fabricated by using an Ogura needle with a curvature radius diameter of 5 micrometers (μm) on a flat electrode with a diameter of 80 mm as the ground plate and a spherical conductor with a diameter of 20 mm as the HV side. The Ogura needle represented a micro-size metal protrusion on the enclosure of the power apparatus. The distance between the spherical HV side electrode and the Ogura needle on the plate ground side electrode was 3 mm. The PD simulator was filled with SF_6_ gas of the gas pressure at 0.5 megapascals (MPa). The upper and bottom covers were made of aluminum alloy (AL-6061), and a gas valve and gas pressure indicator were installed at the bottom cover. A spherical conductor with a diameter of 25 mm was installed to prevent the electric field from concentrating on the high-voltage connection.

### 3.2. Experimental Setup

**Figure 5:**
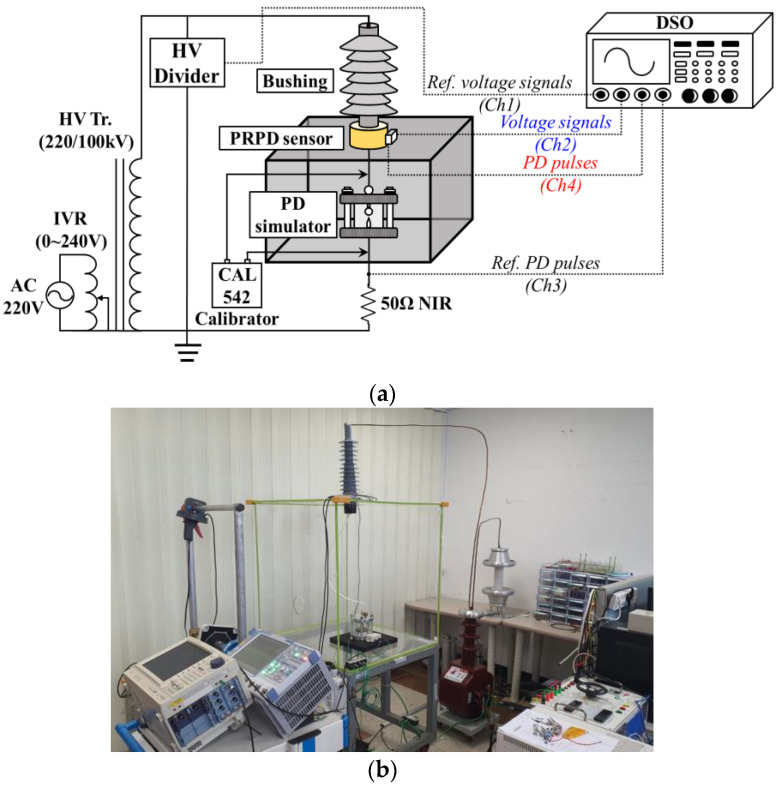
Experimental setup: (**a**) schematic diagram; (**b**) photograph.

Figure 5 shows an experimental setup for evaluating the accuracy of voltage measurement according to IEC 61869-11’s requirements and the PD detection of the prototype PRPD sensor. A dry-type transformer with a maximum output of 100 kV was used to apply a high voltage and was controlled by an induction-type automatic voltage regulator (IVR). An HV divider with an accuracy class of 0.2 and a ratio of 1000:1 was connected to compare the output of the PRPD sensor. A 50 Ω non-inductive resistor (NIR) was installed between the PD simulator and the ground as a conventional electrical detection method according to IEC 60270. All signals from the PRPD sensor, HV divider, and the 50 Ω NIR were recorded using a digital storage oscilloscope (DL9140, YOKOGAWA, Tokyo, Japan), with a sampling rate of 10 megasamples per second (MS/s).

The HV Tr., IVR, HV divider, PD simulator, and measuring devices were grounded to avoid an unexpected electrical potential difference. The level of background noise was less than 3 millivolts (mV) (measured using the prototype PRPD sensor) and 2 mV (measured using a 50 Ω NIR), respectively, in the experiment. To enhance reproducibility, measurements of output accuracy and PD pulses were conducted through experiments repeated more than five times. All parameters were systematically analyzed under consistent conditions to derive reliable results.

## 4. Performance Evaluation

Performance evaluation tests were conducted in two steps: the first was a voltage measurement accuracy test according to the test guidelines in IEC 61869-11; the second was a PD detection test comparing the conventional electrical detection method, according to IEC 60270. Each test step was conducted separately. All performance tests were carried out in a high-voltage laboratory at a room temperature (RT, 23 °C).

### 4.1. Voltage Measurement

**Figure 6:**
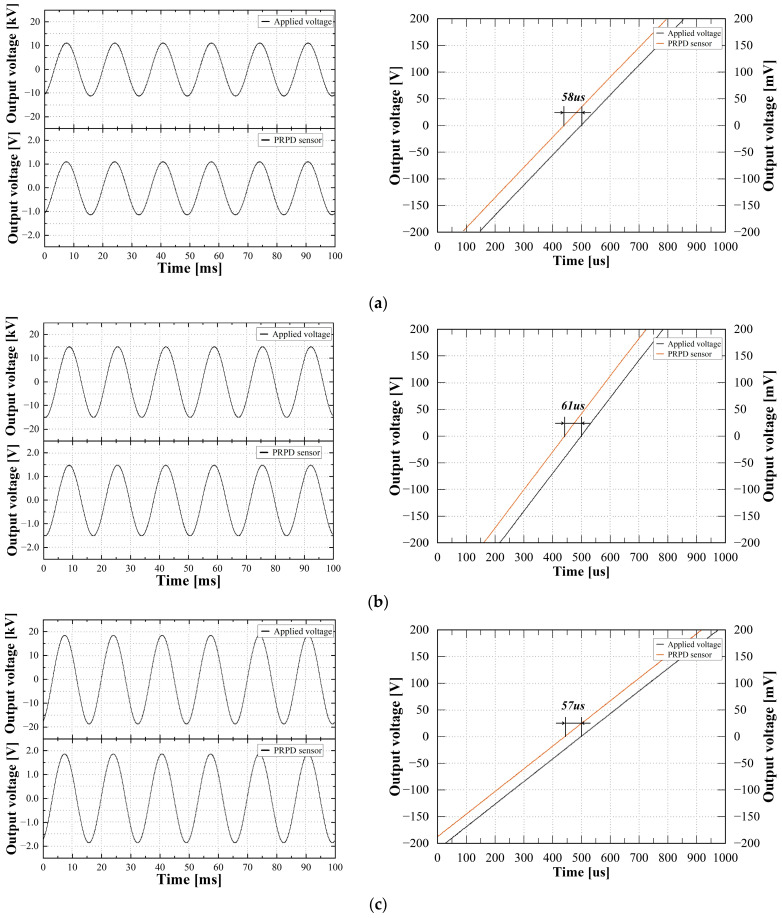
Example waveforms and phase errors: (**a**) 80%; (**b**) 100%; and (**c**) 120%.

To assess the sensitivity and accuracy of the voltage measurement, 80%, 100%, and 120% of the rated voltage (13.2 kV) $U_{P}$ were applied according to the test guideline in IEC 61869-11. Figure 6 shows example waveforms and phase errors of the applied voltage and the PRPD sensor at each voltage level. The voltage waveforms were captured for six cycles to compare the average voltage levels. In addition, the phase errors were confirmed by analyzing each zero-crossing (ZC) point of the waveforms of the applied voltage and the PRPD sensor.

The deviations of the output voltages and phase errors between the applied voltage and the PRPD sensor can be adjusted by the correction factor ${\mathrm{CF}}_{U}$ within a range of 0.900 to 1.100 and phase offset correction $\varphi_{o\;\mathrm{cor}}$ within a range of 300 min (5 degrees (°) or 231 μs). The correction factor ${\mathrm{CF}}_{U}$ and phase offset correction $\varphi_{o\;\mathrm{cor}}$ of the prototype PRPD sensor were set to 1.000 and 76 min, respectively. The corrected voltage ratio error $\varepsilon_{\mathrm{cor}\;U}$ and corrected phase error $\varphi_{e\;\mathrm{cor}}$ were calculated using Equations (3) and (4), respectively:

(3) $$\varepsilon_{\mathrm{cor}\;U}(\%)=\frac{{\mathrm{CF}}_{U}\times K_{r}\times V_{L}-V_{H}}{V_{H}}\times 100$$

(4) $$\varphi_{e\;\mathrm{cor}}=\varphi_{S}-\varphi_{P}-\varphi_{\mathrm{cor}\;\varphi o}$$

where $K_{r}$ is the rated transformation ratio of 10,000, $V_{H}$ is the applied voltage, $V_{L}$ is the output voltage of the PRPD sensor, $\varphi_{S}$ is the phase angle of the PRPD sensor, $\varphi_{P}$ is the phase angle of the applied voltage, and $\varphi_{\mathrm{cor}\;\varphi o}$ is the corrected phase offset.

Table 2 shows the corrected error ratio $\varepsilon_{\mathrm{cor}\;U}$ and corrected phase error $\varphi_{e\;\mathrm{cor}}$ at each applied voltage, i.e., 80%, 100%, and 120% of the rated voltage, calculated using Equations (3) and (4). The maximum values of the voltage ratio error and the phase errors were 0.166% and + 3.06 min, respectively. From the voltage measurement test, the PRPD sensor could meet the accuracy class of 0.2, as specified in IEC 61869-11, because the voltage ratio error and phase error at each applied voltage did not exceed 0.2% and 10 min.

### 4.2. PD Detection

**Figure 7:**
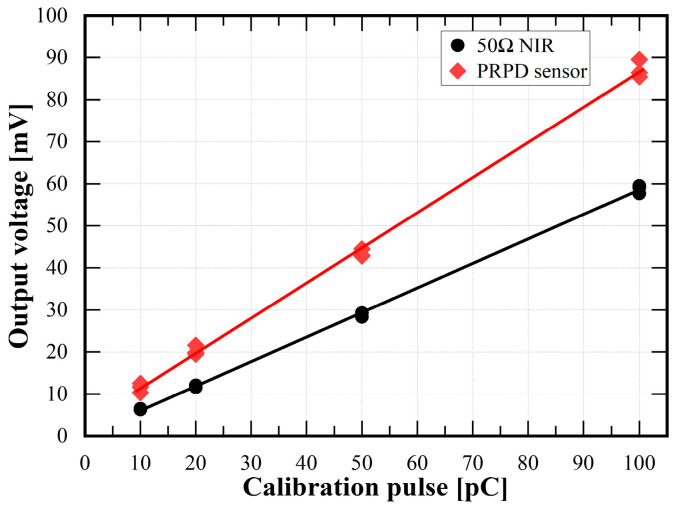
Output voltage for the calibration pulse.

Before the PD detection experiment, a calibration test was conducted to evaluate the linearity of the prototype PRPD sensor using the PD simulator. Artificial calibration pulses of 10 pC, 20 pC, 50 pC, and 100 pC with a rising time of tens of nanoseconds were injected into the PD simulator using a calibrator (CAL 1A, Power Diagnostix Systems GmbH, Aachen, Germany). Figure 7 shows the average output voltage detected by the PRPD sensor and the conventional electrical detection method in accordance with the calibration pulses. Each output voltage of the 50 Ω NIR and PRPD sensor was recorded five times to calculate their average values. The calibration test confirmed that the output of the prototype PRPD sensor had linearity with respect to the calibration inputs and was more sensitive than the conventional method.

**Figure 8:**
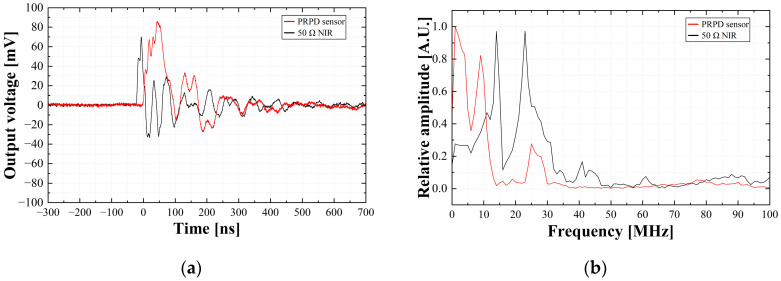
Example waveform: (**a**) single pulse; (**b**) fast Fourier transform (FFT).

Figure 8 shows examples of the single PD pulses from a metal protrusion defect at 4 kV, measured simultaneously with the prototype PRPD sensor and the 50 Ω NIR, and the fast Fourier transform (FFT) results. The rising time, falling time, and pulse width were analyzed by calculating the average values of 10 single PD pulses. They were 47.2 ns, 52.9 ns, and 51.6 ns in the PRPD sensor and 15.8 ns, 13.3 ns, 16.3 ns in the 50 Ω NIR, respectively. The rising and falling time and pulse width of the PRPD sensor were approximately three times longer than those of 50 Ω NIR. The main frequency ranges and maximum frequency with the highest magnitude were distributed from 2 MHz to 10 MHz and 3 MHz in the PRPD sensor, and 13 MHz to 24 MHz and 14 MHz in the 50 Ω NIR, respectively. The frequency spectrums of the PRPD sensor are relatively lower than those of 50 Ω NIR. This is because as the PD pulse flows through the conductor, the UHF component is attenuated by the distributed element. In this case, the frequency ranges over 40 MHz were attenuated.

**Figure 9:**
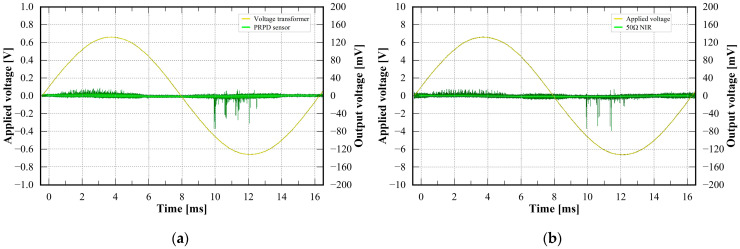
Comparison of PRPD patterns detected by (**a**) PRPD sensor; (**b**) 50 Ω NIR.

Figure 9 shows example PRPD patterns measured by the prototype PRPD sensor and 50 Ω NIR at the same applied voltage of 4 kV. The PD pulses generated from the PD simulator accumulated for 1 min. The applied voltage signal of the prototype PRPD sensor and the 50 Ω NIR was measured by using the VT of the PRPD sensor and HV divider, respectively. The PD pulses of the PRPD sensor were distributed at phase angles of 26° to 105° and 221° to 276°. On the other hand, the PD pulses of the 50 Ω NIR were distributed at phase angles of 25° to 106° and 220° to 269°.

From the comparison of the phase distributions detected by each sensor, the PRPD sensor and 50 Ω NIR, there were no differences between them. In addition, the shape of the PRPD pattern detected by using the prototype PRPD sensor was similar to that of the 50 Ω NIR. According to the PRPD measurement, the prototype PRPD sensor could detect the PRPD patterns phase-synchronized with the applied voltage signals without any additional devices.

## 5. Conclusions

Many PD detection techniques have been extensively studied to diagnose insulation degradation in power equipment, but conventional PD sensors are hampered by the drawback of necessitating an independent device or system for the synchronous detection of PD patterns alongside the applied high-voltage signal. Detecting the PD pulses phase-synchronized with the applied voltage signals is a critical issue due to the PD pulses depending on the magnitude and phase of the applied voltage. This study proposed a novel PRPD sensor embedded in a MV-class bushing which could detect PD pulses phase-synchronized with applied voltage signals for the insulation deterioration diagnosis of electrical power equipment. The prototype PRPD sensor consisted of dual-sensing plates fabricated on the insulated flexible PCB and the signal transducer for calibrating the outputs of the voltage signals and PD pulses. The CVD and D-dot principles were applied to the voltage measurement and PD detection, respectively. In order to assess the efficacy of the suggested PRPD sensor, an experimental system was established. The voltage measurement accuracy of the PRPD sensor was evaluated in accordance with the testing standards specified in IEC 61869-11. Furthermore, the linearity and sensitivity of PD detection were compared with conventional electrical sensing techniques. The experimental results are summarized below:

A. Voltage measurement
  The evaluation of voltage measurement accuracy was focused on the deviation of the output magnitude and phase among the applied voltage and PRPD sensor. The designed rated transformation ratio was 10,000:1. The correction factor and corrected phase offset were set to be 1.000 and 76 min. The maximum corrected error ratio and corrected phase error were 0.126% and +3.06 min, respectively, and they were commonly detected at 100% of the rated voltage.
B. PD detection
  The prototype PRPD sensor was linear to the artificial PD calibration pulses. Alongside that, the outputs of the PRPD sensor were approximately 1.5 times larger than those of the conventional electrical detection method via a 50 Ω NIR. Regarding the time and frequency domains, the rising time of the PD pulse was relatively longer than the falling time, and the maximum magnitude was analyzed in the frequency range of about 24 MHz. The prototype PRPD sensor was able to detect the PRPD patterns phase-synchronized with the applied voltage signal successfully. The phase ranges of the PD pulses detected by the PRPD sensor were almost the same as those detected using the conventional method.

From the experimental results, it is expected that the proposed PRPD sensor holds potential as a viable alternative to conventional PD sensors due to its usefulness in diagnosing internal degradation. However, the PRPD sensor proposed in this paper has the limitation that the PRPD sensor embedded in the MV-class bushing needs to be replaced in terms of installation. Despite these difficulties, once applied, the PRPD sensor is expected to contribute to the continuous PD diagnosis of high-voltage facilities. Furthermore, the PRPD sensor has the potential to be connected to digital interfaces with intelligent electrical devices (IEDs) in line with the transition to digital substations.

**Figure 10:**
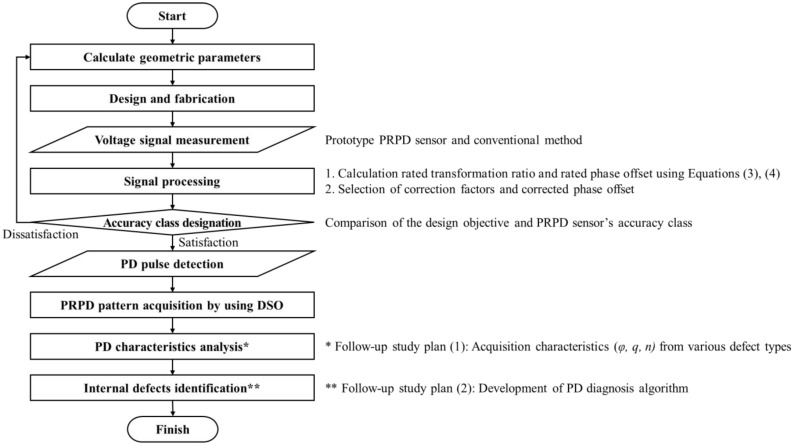
Flowchart of the PRPD measurement method phase-synchronized with applied voltage.

Figure 10 shows a flowchart of the proposed PRPD measurement method phase-synchronized with the applied voltage signal, as proposed in this study. Further research is required as more PD characteristics need to be analyzed for the precise analysis of PD defects. Based on these considerations, additional PD characteristics of various types of PD defects, such as epoxy voids, delamination, cracks, metal suspension, and metal particles in the enclosure, should be investigated, and further research should be conducted on identifying PD defect types and identifying PD sources in the future.

## Figures and Tables

**Table 1:** Geometric parameters of the PRPD sensor.

Parameter		Value
Diameter		Φ 160 mm
Height		12 mm
Sensing electrodes (Voltage and PD)	Width	3 mm
	Thickness	1 ounce (oz)
Insulation layer		0.2 mm
Dielectric constant ($\varepsilon_{s}$)		4.7

**Table 2:** Accuracy test results of the PRPD sensor.

Voltage Level	Applied Voltage $V_{H}$ [kV]	PRPD Sensor $V_{L}$ [V]	Corrected Error Ratio $\varepsilon_{corU}$ [%]		Corrected Phase Error $\varphi_{ecor}$ [min]	
			Measured Value	Accuracy Class of 0.2	Measured Value	Accuracy Class of 0.2
$0.8U_{P}$	10.561	1.056	0.001	0.2	−0.83	10
$1.0U_{P}$	13.204	1.322	0.126		+3.06	
$1.2U_{P}$	15.803	1.583	0.166		−2.13	

## Data Availability

Data are contained within the article.

## Abbreviations and Physical Quantities

The following abbreviations and symbols and expressions of physical quantities are used in this manuscript.

**Abbreviations**		**Full Name**	
PRPD		Phase-resolved partial discharge	
PD		Partial discharge	
PCB		Printed circuit board	
CVD		Capacitive voltage divider	
HFCT		High-frequency current transformer	
UHF		Ultra-high frequency	
AE		Acoustic emission	
S/N		Signal-to-noise ratio	
PT		Potential transformer	
CPT		Capacitive potential transformer	
RPT		Resistive potential transformer	
LPVT		Low-power voltage transformer	
IEC		International Electrotechnical Commission	
SAMU		Stand-alone merging unit	
EVT		Electronic voltage transformer	
TL		Transmission line	
EM		Electromagnetic	
VT		Voltage transformer	
T_g_-type		High glass transition temperature	
GDT		Gas discharge tube	
HPF		High-pass filter	
IVR		Induction-type automatic voltage regulator	
NIR		Non-inductive resistor	
DSO		Digital storage oscilloscope	
HV Tr.		High-voltage transformer	
HV divider		High-voltage divider	
ZC		Zero-crossing	
FFT		Fast Fourier transform	
**Physical quantities**	**Symbols**	**Definitions**	**Units**
Corrected ratio error	$\varepsilon_{\mathrm{cor}\;U}$	Ratio error of an individual passive LPVT corrected by the factor	±%
Correction factor	${\mathrm{CF}}_{U}$	Factor by which the rated transformation ratio evaluated at rated burden and rated frequency of an individual passive LPVT is to be multiplied to achieve the specified accuracy class	-
Rated transformation ratio	$K_{r}$	Ratio of output voltage to the input voltage of the passive LPVT	-
Phase offset correction	$\varphi_{o\;\mathrm{cor}}$	Value to be added to the rated phase offset evaluated at rated burden and rated frequency of an individual passive LPVT to achieve the specified accuracy class	±Minutes ±Centiradians
Corrected phase error	$\varphi_{e\;\mathrm{cor}}$	Phase error of an individual passive LPVT corrected by the value	±Minutes ±Centiradians
Corrected phase offset	$\varphi_{\mathrm{cor}\;\varphi o}$	individual phase offset of a passive LPVT	±Minutes ±Centiradians
